# Supplementary material for: Evolutionary conservation of a core root microbiome across plant phyla along a tropical soil chronosequence
Source: Nat Commun. 2017 Aug 9;8:215. doi: 10.1038/s41467-017-00262-8 (PMC5548757; doi:10.1038/s41467-017-00262-8)
Supplement: Supplementary file 1 — Supplementary Information [file 41467_2017_262_MOESM1_ESM.pdf]

File name: Supplementary Information
Description: Supplementary Figures and Supplementary Tables

File name: Supplementary Data 1
Description: Plant ribulose-bisphosphate carboxylase gene sequences for representing plant phylogeny

File name: Supplementary Software
Description: Software and statistical commands in R for processing and analysis of microbial community data

File name: Peer Review File
Description:

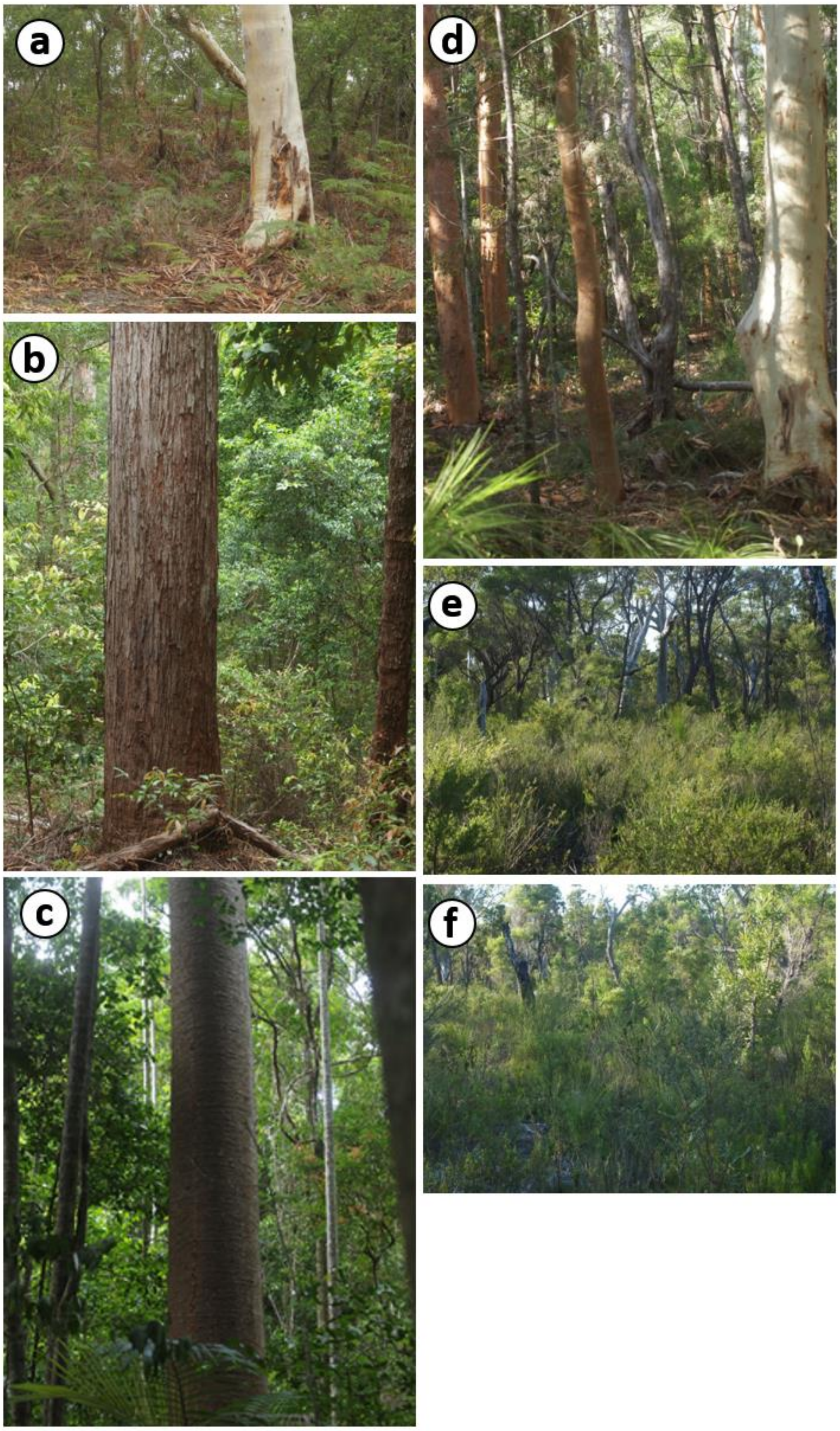

Supplementary Figure 1. Six Cooloola plant communities surveyed. (**a**) Site *a*, open sclerophyll *Eucalyptus racemosa* early successional woodland; (**b**) site *b*, *Eucalyptus pilularis* tall open moist sclerophyll forest; (**c**) site *c*, Rainforest (complex notophyll vine forest) with *Agathis robusta* (main tree in image), *Ficus* spp. and *Archontophoenix cunninghamiana* in fire sheltered parabolic high dunes; (**d**) site *d*, mixed eucalypt conifer open sclerophyll forest with *Eucalyptus racemosa*, *Angophora leiocarpa* and *Callitris rhomboidea*; (**e**) site *e*, retrogression sclerophyll shrubby woodland of *Eucalyptus racemosa*, *Banksia aemula* and *Leptospermum* species; (**f**) site *f*, retrogression Wallum shrubland with *Banksia aemula*, *Xanthorrhoea johnsonii* and a high diversity of heath shrubs.

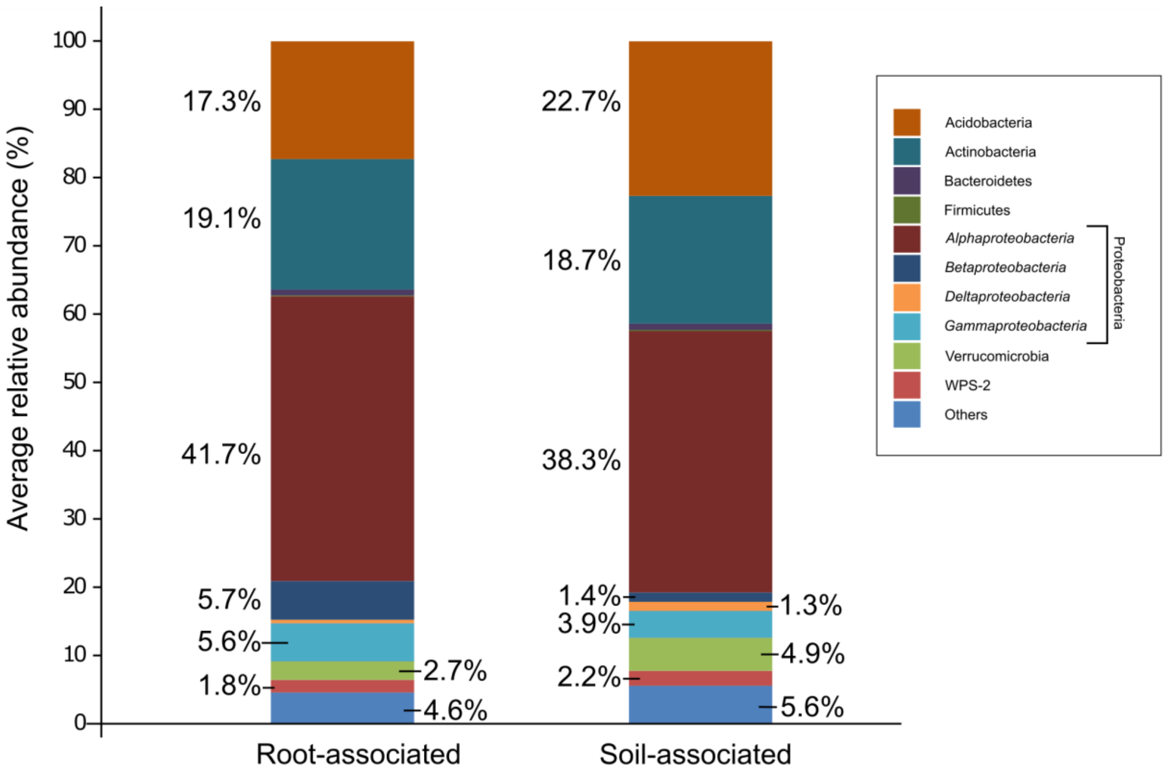

Supplementary Figure 2. Phylum level relative abundance distribution of OTUs root and bulk soil communities. Individual phyla are colour coded according to the legend. Values are indicated for phyla with >1% average relative abundance.

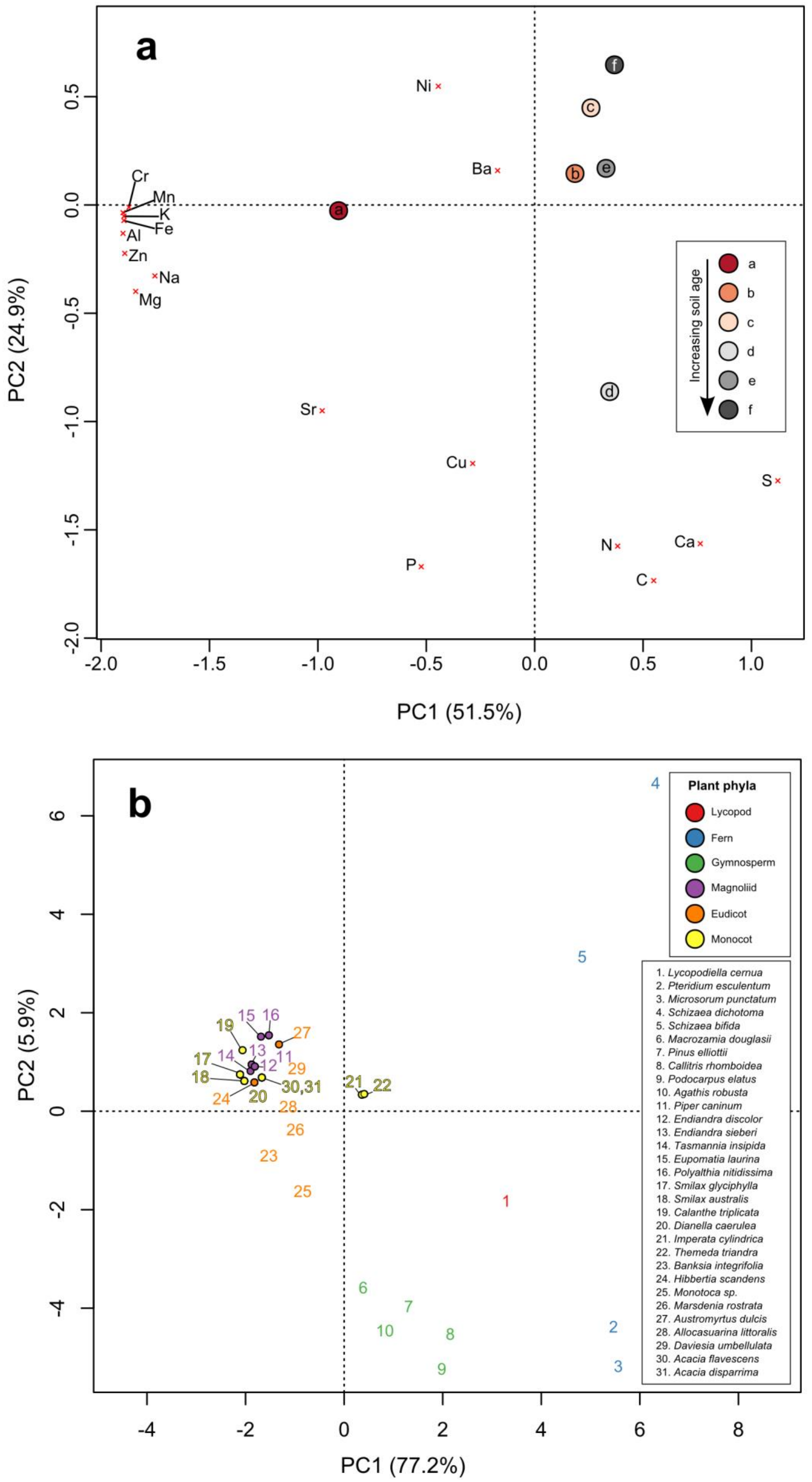

Supplementary Figure 3. Principal component ordination of standardized soil chemical characteristics (**a**), and *rbcL* gene-based plant phylogenetic distances (**b**). Principal component scores were extracted from these principal component ordinations to relate microbial community composition to soil chemical characteristics and host phylogeny.

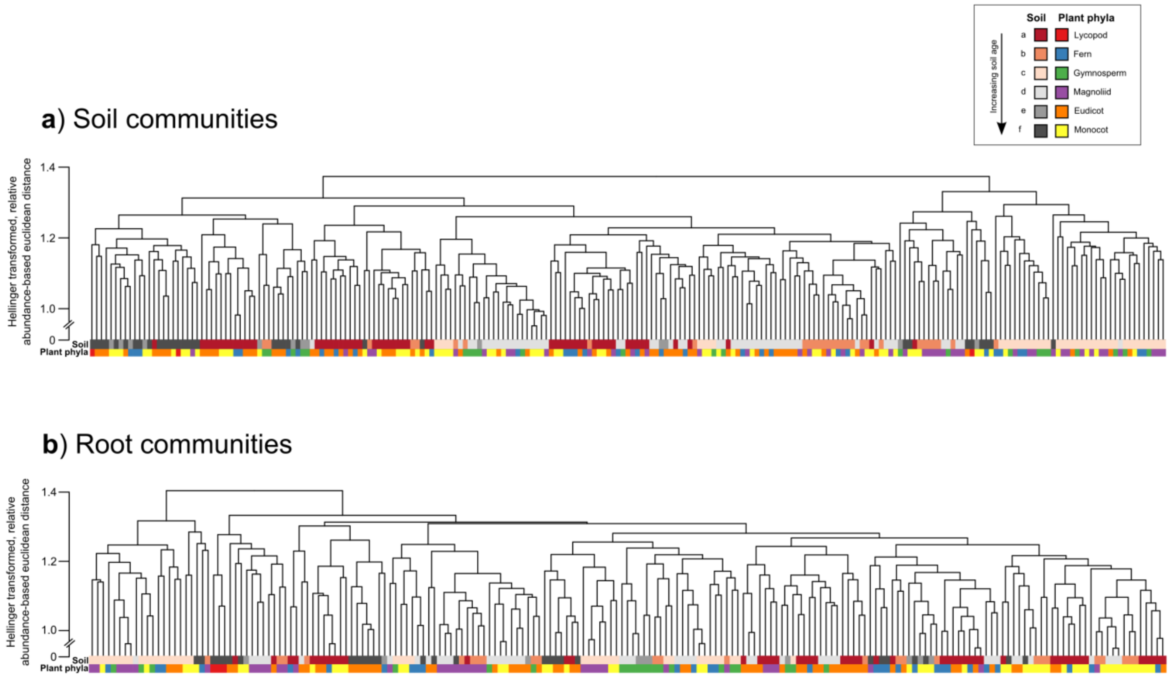

Supplementary Figure 4. Complete linkage hierarchical clustering of (**a**) soil and (**b**) root bacterial communities showing clustering mainly by soil type. Tips are coloured to distinguish between plant community and plant phyla.

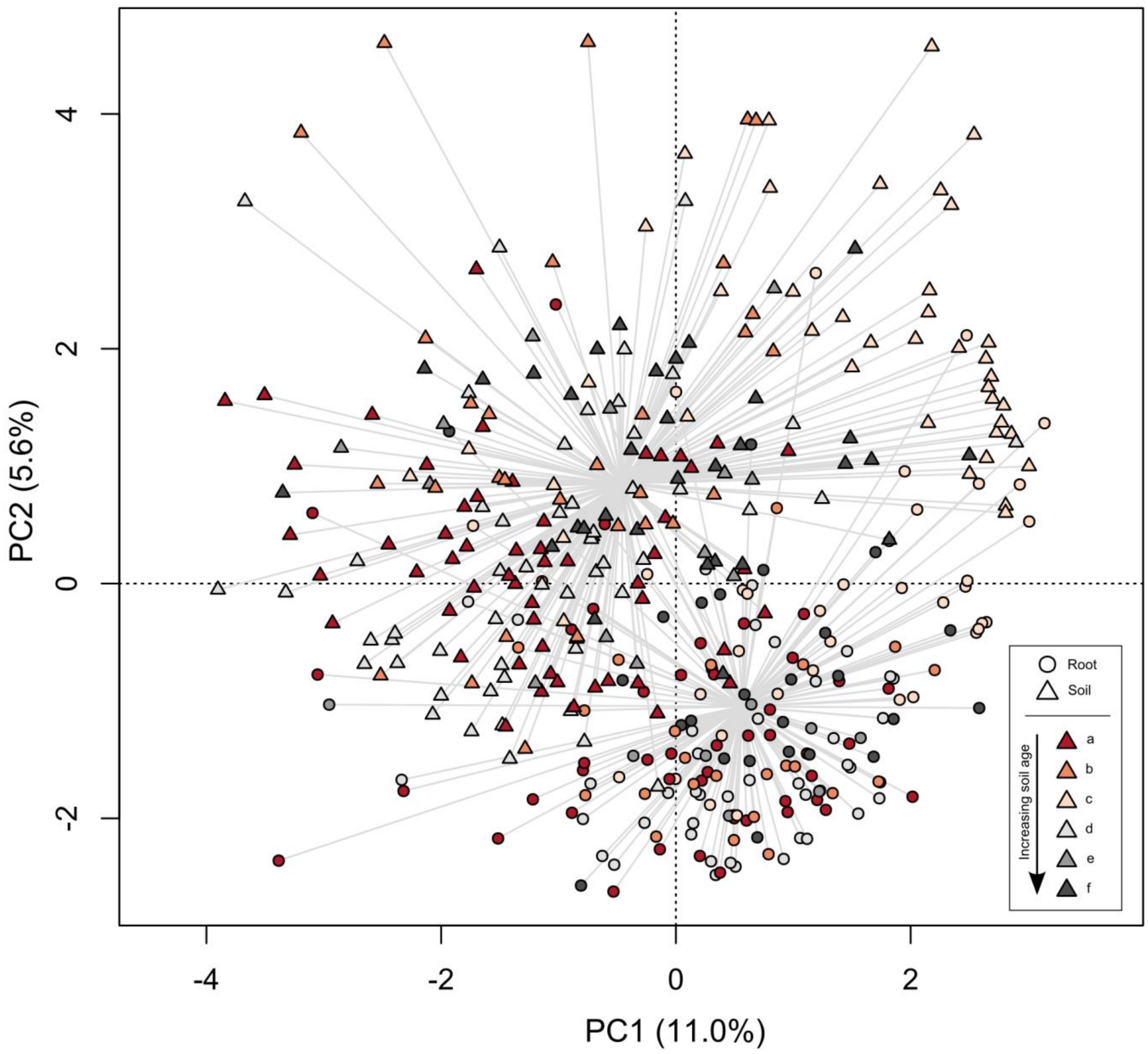

Supplementary Figure 5. Principal component ordination of root and bulk soil bacterial community composition showing relatedness between samples. Each point represents one community (roots as circles, soils as triangles) and is coloured by plant community. All root and bulk soil samples are connected to their respective centroids as indicated by light grey spokes. The root communities significantly differed in composition compared to bulk soils (PERMANOVA, $p < 0.01$).

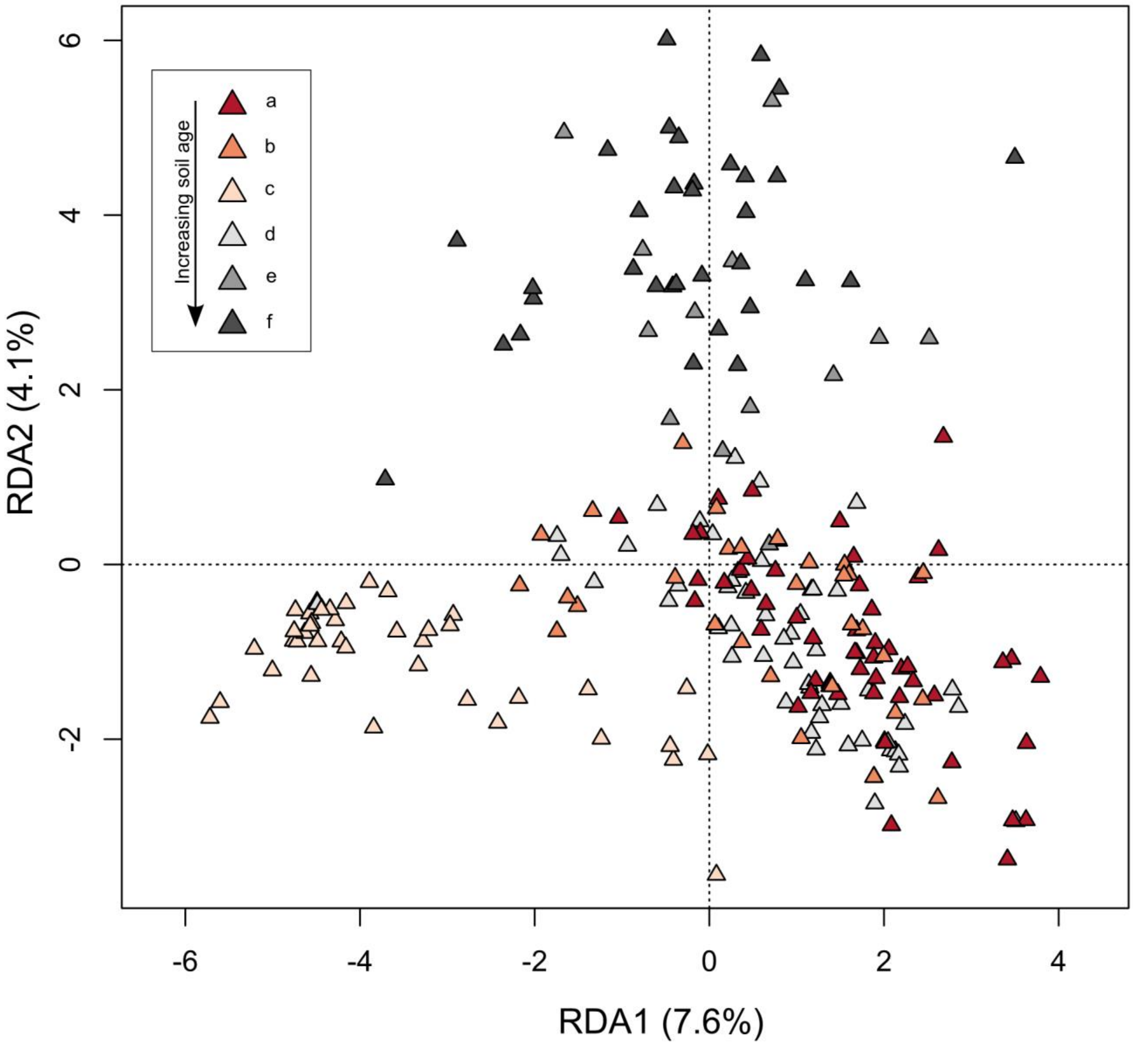

Supplementary Figure 6. Constrained ordination (redundancy analysis) of bulk soil bacterial community composition showing relatedness between samples. Each point represents one bulk soil community and is coloured by plant community. A significant difference in bulk soil bacterial community composition between plant communities was detected ($p < 0.01$, permutation test, 999 permutations).

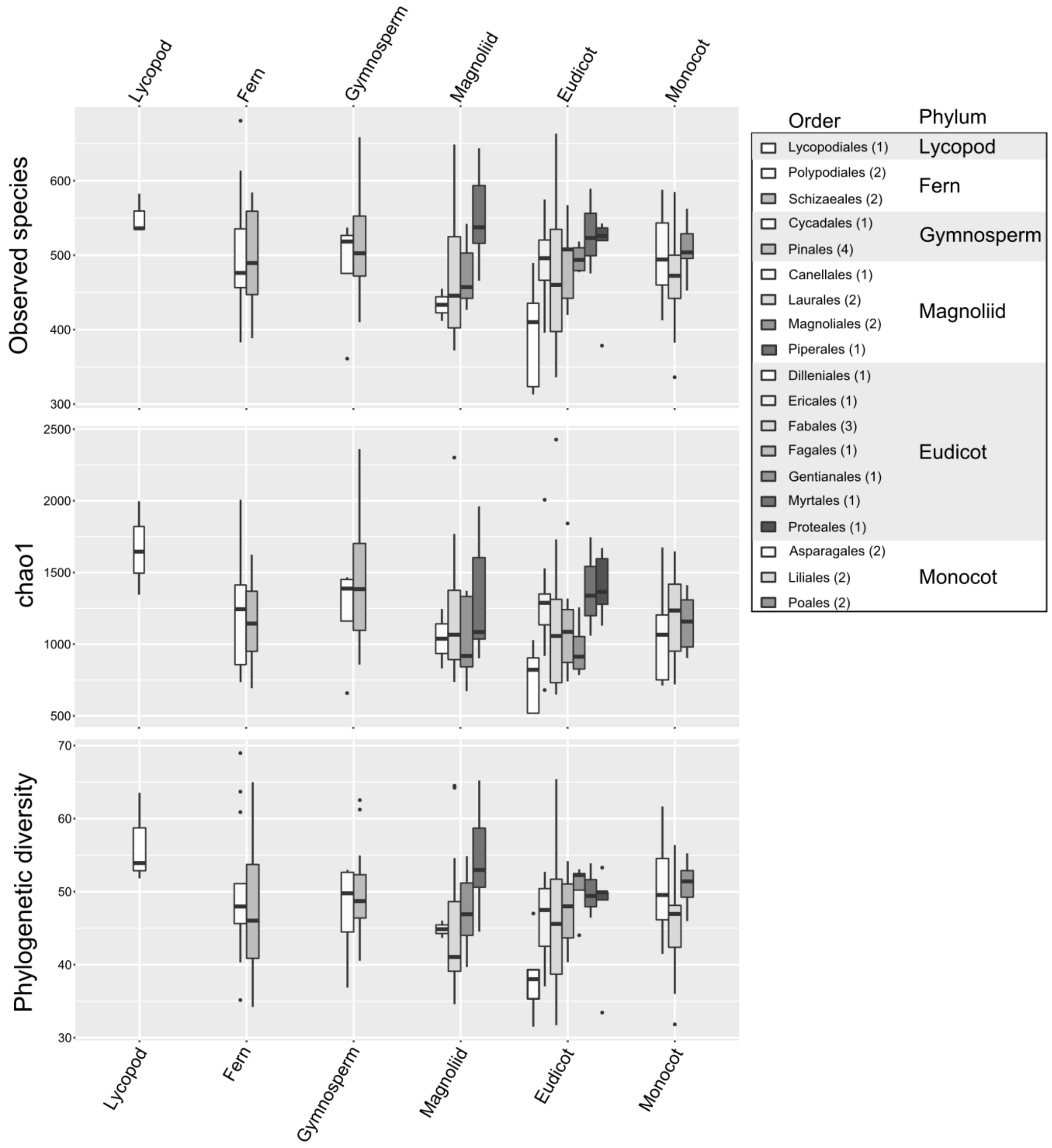

Supplementary Figure 7. Average observed species richness, estimated species richness (chao1) and Faith's phylogenetic diversity of root microbial communities across all plant communities, grouped by plant phyla. The centre line within rectangles represents median values, and the two ends of the rectangle represent upper and lower quartiles. The upper whisker extends to the highest value within 1.5x the interquartile range above the upper quartile, whereas the lower whisker extends to the lowest value within 1.5x the interquartile range below the lower quartile. Values outside this range are represented by black dots. Plant orders are indicated by rectangle colours (see legend, number of species within each order are listed in parenthesis).

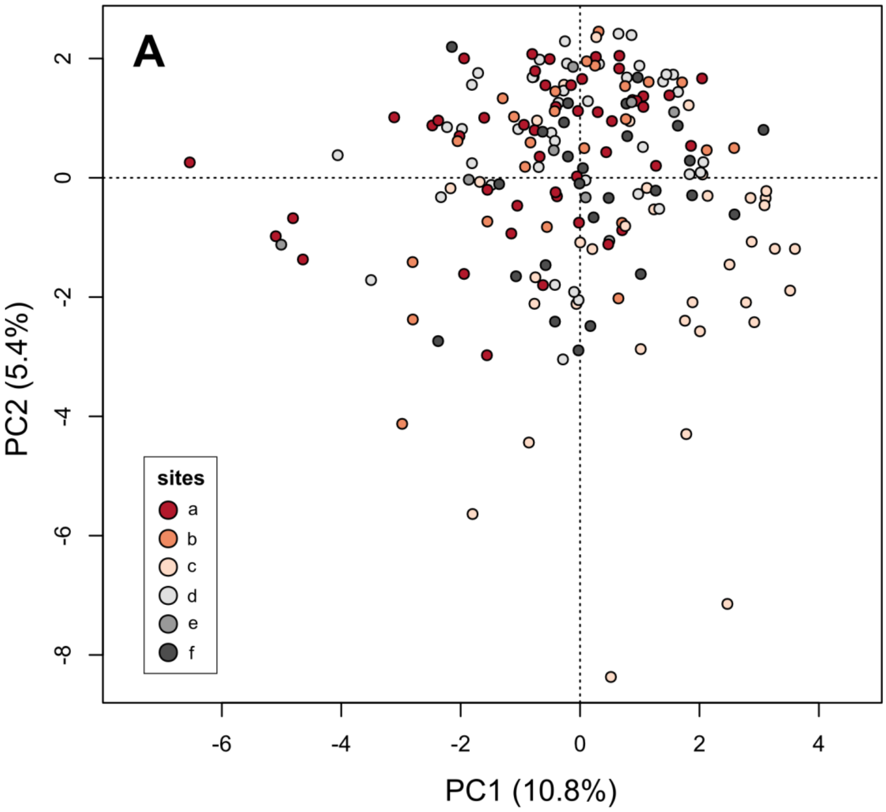

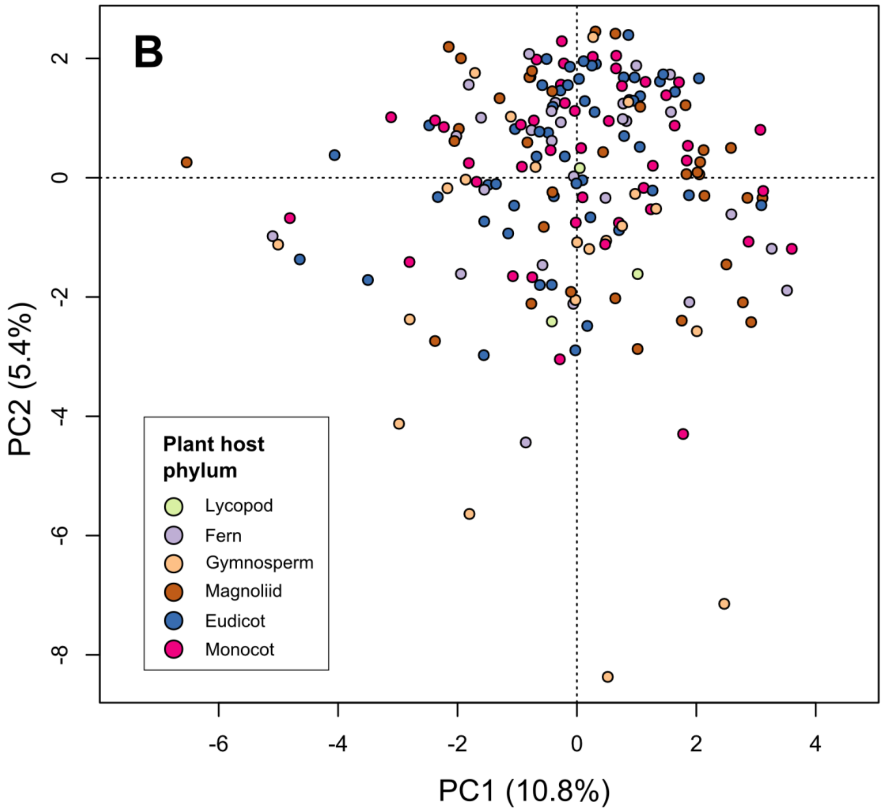

Supplementary Figure 8. Principal component ordination of root bacterial community composition showing relatedness between samples. Each point represents one community and is coloured by plant community (sampling sites) in panel **A** or phylum of their respective plant hosts in panel **B**.

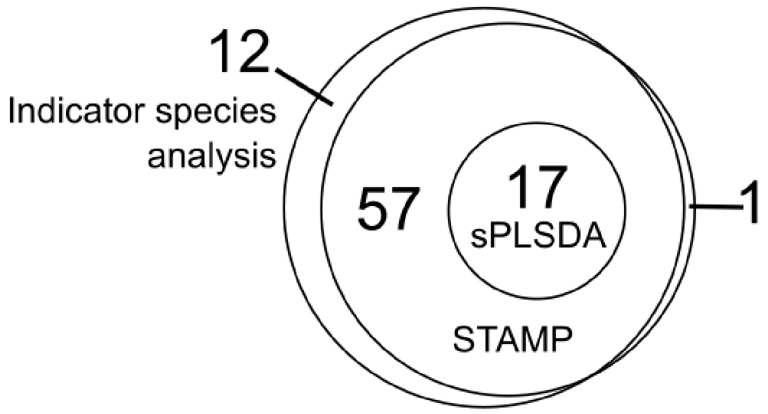

Supplementary Figure 9. Overlap in root-associated bacterial genera between three independent analyses- indicator species analysis, Welch's t-test using STAMP and sparse partial least squares discriminant analysis (sPLSDA) implemented in mixOmics. Values indicate number of taxa significantly associated with Cooloola root microbial communities (see Supplementary Table 6 for list of taxa by analysis method).

Supplementary Table 1. Vegetation type, soil age and type information of the six Cooloola plant communities sampled

Sites	a	b	c	d	e	f
**Vegetation type**	Early-succession sclerophyll woodland	Tall open sclerophyll forest	Rainforest	Mixed eucalypt conifer sclerophyll forest	Retrogression sclerophyll woodland	Retrogression wallum shrubland
**Dune system classification**	DS3	DS4	DS4	DS5	DS6	DS6
**Soil age (years)**	7500 - 9500	130,000 - 170,000	130,000 - 170,000	195,000 - 215,000	280,000 - 395,000	~460,000
**Soil type**	young podzol	podzol	giant podzol	giant podzol	giant humus podzol	giant humus podzol

Supplementary Table 2. Microbial biomass, phosphatase and total enzyme activity, and soil chemical characteristics of bulk soils from the six sampling sites

	Site a	Site b	Site c	Site d	Site e	Site f
FDA activity (µg FDA g^{-1} dry soil h^{-1})	33.5 (1.7)^{BC}	46.3 (2.8)^{AB}	55.7 (2.8)^{A}	58.9 (2.3)^{A}	21.8 (3.7)^{C}	18.2 (4.2)^{C}
Phosphatase activity (µg PNP g^{-1} dry soil h^{-1})	21 (0.3)^{B}	29.3 (2.4)^{A}	19.3(1.1)^{B}	28.9(0.5)^{A}	31 (1.3)^{A}	27.5(1.1)^{A}
pH	4.2 (0.1)^{B}	4.1 (0.1)^{B}	4.6 (0.2)^{A}	4.3 (0.1)^{AB}	4.4 (0.1)^{AB}	4.2 (0.1)^{AB}
Microbial biomass (µg MB-N g^{-1} dry soil)	2.9 (0.2)^{B}	5.3 (0.9)^{AB}	7.8 (1.3)^{A}	5.9 (0.9)^{AB}	2.9 (0.9)^{B}	3.0 (0.5)^{B}
Soil moisture content %	8.7% (1.3)^{A}	8.2% (1.2)^{A}	7.2% (1.0)^{AB}	9.2% (1.4)^{A}	5.6% (1.3)^{B}	4.7% (1.5)^{B}
C wt % dry soil	1.8 (0.4) ^{B}	2.7 (0.7) ^{AB}	1.5 (0.2) ^{B}	3.7 (0.3)^{A}	1.6 (0.3)^{B}	1.4 (0.2)^{B}
N wt %	0.07 (0.008) ^{AB}	0.09 (0.03) ^{AB}	0.08 (0.006) ^{AB}	0.11 (0.02) ^{A}	0.06 (0.01) ^{AB}	0.04 (0.006)^{B}
Al mg kg^{-1} dry soil	3137 (197) ^{A}	480 (63) ^{B}	378 (60) ^{B}	413 (8) ^{B}	350 (51) ^{B}	168 (13) ^{B}
Ba mg kg^{-1}	34 (5)	40 (6)	43 (6)	32 (10)	22 (3)	25 (1)
Ca mg kg^{-1}	319 (8)	379 (109)	325 (31)	471 (155)	262 (45)	333 (35)
Cr mg kg^{-1}	59 (9) ^{A}	16 (3) ^{B}	11 (3) ^{B}	14 (3) ^{B}	11(3) ^{B}	19 (1) ^{B}
Cu mg kg^{-1}	2.8 (0.6)	2.7 (0.5)	1.5 (0.2)	3.1 (0.8)	4.1 (1.6)	2.1 (0.2)
Fe mg kg^{-1}	6818 (1087) ^{A}	187 (30) ^{B}	156 (26) ^{B}	195 (29) ^{B}	99 (12) ^{B}	100 (13) ^{B}
K mg kg^{-1}	1482 (180) ^{A}	386 (135) ^{B}	77 (11) ^{B}	72 (5.5) ^{B}	93 (30) ^{B}	9.4 (0.7) ^{B}
Mg mg kg^{-1}	248 (21) ^{A}	123 (31) ^{B}	92 (18) ^{B}	109 (25) ^{B}	60 (3) ^{B}	54 (5) ^{B}
Mn mg kg^{-1}	289 (38) ^{A}	19 (2) ^{B}	25 (4) ^{B}	13 (2) ^{B}	7 (1) ^{B}	9 (1) ^{B}
Na mg kg^{-1}	606 (50) ^{A}	346 (22) ^{B}	267 (32) ^{B}	235 (35) ^{B}	97 (17) ^{C}	43 (6) ^{C}
Ni mg kg^{-1}	22 (2)	23 (3)	26 (2)	19 (6)	14 (2)	18 (1)
P mg kg^{-1}	95 (8) ^{AB}	66 (13) ^{BC}	75 (13) ^{AB}	119 (14) ^{A}	47 (13) ^{BC}	26 (4) ^{C}
S mg kg^{-1}	183 (10) ^{C}	155 (21) ^{C}	392 (50) ^{B}	671 (48) ^{A}	461 (58) ^{B}	293 (30) ^{BC}
Sr mg kg^{-1}	21 (2)	6.0 (2)	10 (3)	19 (6)	18 (5)	15 (2)
Zn mg kg^{-1}	19 (1) ^{A}	6 (1) ^{B}	5 (1) ^{B}	6 (1) ^{B}	5 (2) ^{B}	4 (1) ^{B}

Data is the means of five replicates from each site. Each replicate was pooled from 3-10 soil random soil samples at each site. Standard error of the mean is given in brackets. Statistics were performed using one-way ANOVA with Tukey's post-test. ($p < 0.05$). Superscript alphabets denote statistically significant groups of measurement values. Fluorescein diacetate hydrolysis (FDA) is used to determine total microbial activity levels. Microbial biomass was assessed using chloroform fumigation/extraction then a ninhydrin assay for N content. Carbon and nitrogen data by combustion (Dumas) protocol using a LECO TruSpec analyser; other elements by microwave digestion and analysis using a Varian Vista Pro ICPOES instrument

Supplementary Table 3. Cooloola core root microbiome taxa based on indicator species analysis on total sum scaled OTU relative abundances

	Taxonomy						Average relative abundance (%)								Also core in:				
	Phylum	Class	Order	Family	Genus	Number of OTUs represented	Lycopod	Ferns	Gymno-sperms	Magnoliids	Eudicots	Monocots	All roots	All soils	sugarcane (Australia)	*A. thaliana* (Germany)	Barley (Germany)	Grapevine (America)	Maize (America)
OTUs with genus classification	*Actinobacteria*	*Actinobacteria*	*Actinomycetales*	*Actinospicaceae*	*Actinospica*	2	0.00	0.27	0.26	0.58	0.54	0.26	0.40	0.04					
				Actinosynnemataceae	*Kutzneria*	1	0.05	0.11	0.07	0.09	0.09	0.10	0.09	0.03					
				Mycobacteriaceae	*Mycobacterium*	7	0.02	0.19	0.29	0.17	0.36	0.36	0.28	0.09					
				Streptomycetaceae	*Streptomyces*	2	0.47	0.10	0.03	0.08	0.02	0.06	0.06	0.02	Y	Y	Y	Y	Y
	Armatimonadetes	*Fimbriimonadia*	*Fimbriimonadales*	*Fimbriimonadaceae*	*Fimbriimonas*	1	0.10	0.03	0.06	0.10	0.04	0.05	0.06	0.02	Y			Y	
	Proteobacteria	*Alphaproteobacteria*	*Caulobacterales*	*Caulobacteraceae*	*Asticcacaulis*	3	0.46	0.28	0.16	0.47	0.12	0.25	0.25	0.03	Y				
			Rhizobiales	*Beijerinckiaceae*	*Methylocapsa*	2	0.00	0.07	0.50	0.07	0.13	0.12	0.15	0.04					
				Bradyrhizobiaceae	*Afipia*	1	0.00	0.05	0.01	0.47	0.24	0.28	0.24	0.00	Y				
					Bradyrhizobium	10	1.73	4.58	6.24	5.96	4.70	4.94	5.12	3.09	Y			Y	
				Hyphomicrobiaceae	*Rhodoplanes*	7	0.27	0.79	0.44	1.18	1.04	0.74	0.87	0.32				Y	
				Phyllobacteriaceae	*Mesorhizobium*	1	0.01	0.09	0.16	0.30	0.05	0.08	0.12	0.03	Y	Y	Y		Y
					Nitratireductor	1	0.00	0.00	0.00	0.14	0.02	0.02	0.04	0.00					
				Rhizobiaceae	*Agrobacterium*	1	0.00	0.05	0.15	0.10	0.15	0.32	0.16	0.01	Y	Y	Y	Y	Y
					Rhizobium	2	0.38	0.07	0.45	0.82	0.07	0.25	0.31	0.01	Y	Y	Y	Y	Y
					Shinella	1	0.03	0.00	0.43	0.04	0.02	0.09	0.09	0.00					
				Xanthobacteraceae	*Labrys*	3	0.53	0.12	0.29	0.23	0.03	0.13	0.14	0.03					
			Rhodospirillales	*Acetobacteraceae*	*Acidisoma*	4	0.27	0.13	0.24	0.29	0.31	0.14	0.23	0.06					
					Acidocella	2	0.00	0.02	0.12	0.02	0.56	0.05	0.19	0.01					
				Rhodospirillaceae	*Azospirillum*	6	0.01	0.22	1.58	0.80	0.31	0.34	0.54	0.01					
					Telmatospirillum	3	1.82	0.33	0.31	1.35	0.35	0.42	0.58	0.11	Y				
			Sphingomonadales	*Sphingomonadaceae*	*Sphingomonas*	1	0.27	0.02	0.18	0.14	0.10	0.05	0.10	0.02	Y	Y	Y	Y	Y
		Betaproteobacteria	*Burkholderiales*	*Burkholderiaceae*	*Burkholderia*	34	7.25	1.26	1.79	2.29	2.25	3.93	2.55	0.29	Y				Y
					Salinispora	5	0.95	0.19	0.32	0.25	0.24	0.34	0.28	0.07					
				Comamonadaceae	*Rubrivivax*	2	0.34	0.06	0.13	0.03	0.08	0.06	0.07	0.00		Y	Y		Y
				Oxalobacteraceae	*Cupriavidus*	3	0.00	0.00	0.00	0.04	0.11	0.08	0.06	0.00	Y				
		Gammaproteobacteria	*Xanthomonadales*	*Xanthomonadaceae*	*Dyella*	5	0.12	0.31	0.13	0.89	0.43	1.84	0.80	0.01	Y				
					Luteibacter	1	0.00	0.00	0.00	0.02	0.01	0.19	0.05	0.00	Y				
					Rhodanobacter	2	0.06	0.01	0.01	0.02	0.04	0.12	0.05	0.00	Y				
	Tenericutes	*Mollicutes*	*Anaeroplasmatales*	*Anaeroplasmataceae*	*Asteroleplasma*	9	0.00	0.02	0.26	0.13	0.07	0.20	0.13	0.00					

Supplementary Table 3 continued – OTUs without genus classification

	Taxonomy						Average relative abundance (%)								Also core in:				
	Phylum	Class	Order	Family	Genus	Number of OTUs represented	Lycopod	Ferns	Gymno-sperms	Magnoliids	Eudicots	Monocots	All roots	All soils	sugarcane (Australia)	*A. thaliana* (Germany)	Barley (Germany)	Grapevine (America)	Maize (America)
OTUs without genus classification	*Acidobacteria*	*Acidobacteriia*	*Acidobacteriales*	*Acidobacteriaceae*	-	10	4.25	3.73	3.62	2.63	3.59	2.91	3.27	1.49	Y				
				Koribacteraceae	-	3	0.00	0.04	0.01	0.25	0.18	0.24	0.16	0.00				Y	
	Actinobacteria	*Actinobacteria*	*Actinomycetales*	-	-	27	0.30	1.41	2.27	1.38	1.97	0.91	1.52	0.38	Y				
				Actinospicaceae	-	14	0.07	1.38	1.22	1.03	1.74	1.32	1.36	0.06	Y				
				Micromonosporaceae	-	3	0.05	0.15	0.12	0.13	0.01	0.13	0.09	0.02	Y			Y	
				Pseudonocardiaceae	-	2	0.00	0.01	0.18	0.08	0.09	0.04	0.07	0.02	Y				
				Thermomonosporaceae	-	1	0.00	0.01	0.12	0.03	0.00	0.01	0.02	0.00				Y	
		Thermoleophilia	*Solirubrobacterales*	*Conexibacteraceae*	-	4	0.01	0.16	0.12	0.18	0.47	0.18	0.25	0.08					
	Armatimonadetes	*Armatimonadia*	*FW68*	-	-	3	0.03	0.14	0.08	0.03	0.04	0.09	0.07	0.00					
	Bacteroidetes	*Saprospirae*	*Saprospirales*	*Chitinophagaceae*	-	9	0.81	0.42	0.42	0.52	0.23	0.40	0.39	0.08	Y			Y	Y
	Chloroflexi	*Ktedonobacteria*	*JG30-KF-AS9*	-	-	1	0.08	0.05	0.14	0.02	0.07	0.04	0.06	0.01					
		Ktedonobacteria	*Ktedonobacterales*	*Ktedonobacteraceae*	-	13	0.00	0.20	0.29	0.02	0.61	0.18	0.28	0.01	Y				
		Ktedonobacteria	*Thermogemmatisporales*	*Thermogemmatisporaceae*	-	1	0.00	0.04	0.55	0.02	0.07	0.01	0.10	0.00	Y				
	Planctomycetes	*Phycisphaerae*	*WD2101*	-	-	1	0.01	0.08	0.10	0.03	0.05	0.02	0.05	0.02					
	Proteobacteria	*Alphaproteobacteria*	*Caulobacterales*	*Caulobacteraceae*	-	6	0.90	0.10	0.05	0.16	0.35	0.28	0.23	0.05	Y				
			Ellin329	-	-	1	0.00	0.04	0.08	0.10	0.09	0.03	0.07	0.03				Y	
			Rhizobiales	-	-	5	0.22	0.10	0.17	0.23	0.08	0.17	0.15	0.01	Y		Y	Y	
				Beijerinckiaceae	-	2	0.14	0.11	0.18	0.13	0.07	0.21	0.14	0.02					
				Bradyrhizobiaceae	-	5	0.10	0.32	1.26	0.43	0.42	0.78	0.59	0.22					
				Hyphomicrobiaceae	-	1	0.00	0.12	0.03	0.00	0.00	0.00	0.02	0.00				Y	
				Methylocystaceae	-	10	1.66	3.04	2.40	2.63	2.93	2.87	2.79	1.39					
				Rhizobiaceae	-	2	0.22	0.17	0.49	0.27	0.04	0.32	0.23	0.04		Y	Y		
			Rhodospirillales	*Acetobacteraceae*	-	23	1.54	0.70	0.83	1.55	1.16	0.89	1.07	0.31					
				Rhodospirillaceae	-	33	4.53	2.32	3.12	3.26	2.31	2.34	2.63	0.79	Y			Y	
		Betaproteobacteria	*Burkholderiales*	*Burkholderiaceae*	-	3	2.75	0.17	0.16	0.49	1.09	1.24	0.79	0.03	Y				
				Comamonadaceae	-	5	0.21	0.03	0.39	0.31	0.10	0.28	0.21	0.02	Y	Y	Y	Y	Y
		Gammaproteobacteria	*Xanthomonadales*	*Sinobacteraceae*	-	13	1.52	0.68	1.50	1.49	1.42	1.22	1.29	0.19	Y			Y	Y
				Xanthomonadaceae	-	7	0.28	0.30	0.26	0.28	0.31	0.45	0.33	0.04	Y	Y	Y	Y	
	TM7	*TM7-1*	-	-	-	4	0.00	0.10	0.01	0.09	0.08	0.06	0.07	0.01	Y				
	Verrucomicrobia	*Methylacidiphilae*	*Methylacidiphilales*	-	-	2	0.51	0.04	0.12	0.07	0.04	0.04	0.06	0.02	Y				
	WPS-2	-	-	-	-	2	0.07	0.17	0.36	0.73	0.34	0.38	0.40	0.16					
					Sum:	338	35.4	25.7	35.3	35.6	32.4	33.9	32.8	9.9					
					Sum including low abundance OTUs:	369	35.5	26.0	35.6	36.3	33.0	34.2	33.2	9.9					

- Based on indicator species analysis on total sum scaled OTU relative abundances
- Relative abundances are shaded by column from white to red representing lowest to highest values in the respective plant phyla.
- "Y" represents "yes" for the five rightmost columns under "Also core in:"

Supplementary Table 4. Cooloola core root microbiome taxa based on STAMP on total sum scaled OTU relative abundances

	Taxonomy						Average relative abundance (%)							
	Phylum	Class	Order	Family	Genus	Number of OTUs represented	Lycopod	Ferns	Gymno-sperms	Magnoliids	Eudicots	Monocots	All roots	All soils
OTUs with genus classification	*Actinobacteria*	*Actinobacteria*	*Actinomycetales*	*Actinospicaceae*	*Actinospica*	2	0.00	0.27	0.26	0.58	0.54	0.26	0.40	0.04
				Actinosynnemataceae	*Kutzneria*	1	0.05	0.11	0.07	0.09	0.09	0.10	0.09	0.03
				Mycobacteriaceae	*Mycobacterium*	6	0.02	0.19	0.29	0.17	0.34	0.35	0.28	0.09
				Streptomycetaceae	*Streptomyces*	1	0.47	0.08	0.03	0.06	0.02	0.05	0.05	0.02
	Armatimonadetes	*Fimbriimonadia*	*Fimbriimonadales*	*Fimbriimonadaceae*	*Fimbriimonas*	1	0.10	0.03	0.06	0.10	0.04	0.05	0.06	0.02
	Proteobacteria	*Alphaproteobacteria*	*Caulobacterales*	*Caulobacteraceae*	*Asticcacaulis*	3	0.46	0.28	0.16	0.47	0.12	0.25	0.25	0.03
			Rhizobiales	*Bradyrhizobiaceae*	*Afipia*	1	0.00	0.05	0.01	0.47	0.24	0.28	0.24	0.00
					Bradyrhizobium	9	0.71	0.98	1.98	1.62	1.19	1.35	1.36	0.39
				Hyphomicrobiaceae	*Rhodoplanes*	5	0.27	0.75	0.44	1.17	1.02	0.73	0.86	0.32
				Phyllobacteriaceae	*Mesorhizobium*	1	0.01	0.09	0.16	0.30	0.05	0.08	0.12	0.03
					Nitratireductor	1	0.00	0.00	0.00	0.14	0.02	0.02	0.04	0.00
				Rhizobiaceae	*Agrobacterium*	1	0.00	0.05	0.15	0.10	0.15	0.32	0.16	0.01
					Rhizobium	1	0.38	0.07	0.39	0.81	0.03	0.20	0.28	0.00
					Shinella	1	0.03	0.00	0.43	0.04	0.02	0.09	0.09	0.00
				Xanthobacteraceae	*Labrys*	3	0.53	0.12	0.29	0.23	0.03	0.13	0.14	0.03
			Rhodospirillales	*Acetobacteraceae*	*Acidisoma*	4	0.27	0.13	0.24	0.29	0.31	0.14	0.23	0.06
				Rhodospirillaceae	*Azospirillum*	6	0.01	0.22	1.58	0.80	0.31	0.34	0.54	0.01
					Telmatospirillum	3	1.82	0.33	0.31	1.35	0.35	0.42	0.58	0.11
			Sphingomonadales	*Sphingomonadaceae*	*Sphingomonas*	2	0.42	0.04	0.25	0.17	0.11	0.07	0.12	0.03
		Betaproteobacteria	*Burkholderiales*	*Burkholderiaceae*	*Burkholderia*	31	7.33	1.34	1.84	2.40	2.27	3.97	2.60	0.33
					Salinispora	4	0.88	0.19	0.30	0.25	0.15	0.30	0.24	0.06
				Comamonadaceae	*Rubrivivax*	2	0.34	0.06	0.13	0.03	0.08	0.06	0.07	0.00
		Gammaproteobacteria	*Xanthomonadales*	*Xanthomonadaceae*	*Dyella*	5	0.12	0.31	0.13	0.89	0.43	1.84	0.80	0.01
					Rhodanobacter	2	0.06	0.01	0.01	0.02	0.04	0.12	0.05	0.00
	Tenericutes	*Mollicutes*	*Anaeroplasmatales*	*Anaeroplasmataceae*	*Asteroleplasma*	5	0.00	0.02	0.26	0.11	0.05	0.11	0.09	0.00

Supplementary Table 4 continued – OTUs without genus classification

	Taxonomy						Average relative abundance (%)							
	Phylum	Class	Order	Family	Genus	Number of OTUs represented	Lycopod	Ferns	Gymno-sperms	Magnoliids	Eudicots	Monocots	All roots	All soils
OTUs without genus classification	*Acidobacteria*	*Acidobacteriia*	*Acidobacteriales*	*Acidobacteriaceae*	-	12	4.25	3.82	3.67	2.77	3.64	2.95	3.34	1.51
			Acidobacteriales	*Koribacteraceae*	-	3	0.00	0.04	0.01	0.25	0.18	0.24	0.16	0.00
	Actinobacteria	*Actinobacteria*	*Actinomycetales*	-	-	20	0.30	1.34	1.73	1.36	1.90	0.87	1.42	0.38
				Actinospicaceae	-	11	0.07	1.38	1.22	0.92	1.74	1.29	1.33	0.06
				Micromonosporaceae	-	1	0.05	0.14	0.12	0.10	0.01	0.09	0.08	0.02
				Pseudonocardiaceae	-	2	0.00	0.01	0.20	0.24	0.10	0.14	0.13	0.04
				Thermomonosporaceae	-	1	0.00	0.01	0.12	0.03	0.00	0.01	0.02	0.00
		Thermoleophilia	*Solirubrobacterales*	*Conexibacteraceae*	-	4	0.01	0.16	0.12	0.18	0.47	0.18	0.25	0.08
	Armatimonadetes	*Armatimonadia*	*FW68*	-	-	3	0.03	0.14	0.08	0.03	0.04	0.09	0.07	0.00
	Bacteroidetes	*Saprospirae*	*Saprospirales*	*Chitinophagaceae*	-	7	0.80	0.34	0.41	0.50	0.23	0.39	0.37	0.08
	Chloroflexi	*Ktedonobacteria*	*JG30-KF-AS9*	-	-	1	0.08	0.05	0.14	0.02	0.07	0.04	0.06	0.01
			Ktedonobacterales	*Ktedonobacteraceae*	-	5	0.00	0.14	0.21	0.02	0.42	0.13	0.20	0.01
	Gemmatimonadetes	*Gemmatimonadetes*	-	-	-	1	0.00	0.04	0.13	0.02	0.06	0.04	0.05	0.02
	Planctomycetes	*Phycisphaerae*	*WD2101*	-	-	1	0.01	0.08	0.10	0.03	0.05	0.02	0.05	0.02
	Proteobacteria	*Alphaproteobacteria*	*Caulobacterales*	*Caulobacteraceae*	-	5	0.19	0.07	0.16	0.16	0.15	0.16	0.14	0.04
			Ellin329	-	-	1	0.00	0.04	0.08	0.10	0.09	0.03	0.07	0.03
			Rhizobiales	-	-	4	0.22	0.10	0.17	0.21	0.07	0.16	0.14	0.01
				Beijerinckiaceae	-	2	0.14	0.11	0.18	0.13	0.07	0.21	0.14	0.02
				Bradyrhizobiaceae	-	5	0.10	0.32	1.26	0.43	0.42	0.78	0.59	0.22
				Methylocystaceae	-	10	0.50	1.58	1.69	1.65	1.70	1.84	1.68	0.51
				Rhizobiaceae	-	2	0.22	0.17	0.49	0.27	0.04	0.32	0.23	0.04
			Rhodospirillales	*Acetobacteraceae*	-	20	0.53	0.75	1.79	1.60	1.33	0.91	1.24	0.36
				Rhodospirillaceae	-	30	4.35	2.43	3.49	3.25	2.41	2.39	2.73	0.84
		Betaproteobacteria	*Burkholderiales*	*Burkholderiaceae*	-	3	2.75	0.17	0.16	0.49	1.09	1.24	0.79	0.03
				Comamonadaceae	-	3	0.21	0.03	0.30	0.13	0.10	0.26	0.16	0.02
		Gammaproteobacteria	*Xanthomonadales*	*Sinobacteraceae*	-	11	1.51	0.66	1.50	1.46	1.42	1.22	1.28	0.19
				Xanthomonadaceae	-	6	0.39	0.31	0.29	0.38	0.28	0.48	0.35	0.05
	Verrucomicrobia	*Methylacidiphilae*	*Methylacidiphilales*	-	-	2	0.51	0.04	0.12	0.07	0.04	0.04	0.06	0.02
	WPS-2	-	-	-	-	2	0.07	0.18	0.34	0.79	0.38	0.39	0.42	0.18
					Sum:	279	31.6	20.4	30.1	30.2	26.5	28.5	27.3	6.4
					Sum including low abundance OTUs:	302	31.6	20.7	30.4	30.8	27.0	29.1	27.8	6.5

- Based on STAMP on total sum scaled OTU relative abundances
- Relative abundances are shaded by column from white to red representing lowest to highest values in the respective plant phyla.

Supplementary Table 5. Cooloola core root microbiome taxa based on sPLSDA

	Taxonomy						Average relative abundance (%)							
	Phylum	Class	Order	Family	Genus	Number of OTUs represented	Lycopod	Ferns	Gymnosper	Magnoliids	Eudicots	Monocots	All roots	All soils
OTUs with genus classification	*Proteobacteria*	*Alphaproteobacteria*	*Rhizobiales*	*Bradyrhizobiaceae*	*Afipia*	1	0.00	0.05	0.01	0.47	0.24	0.28	0.24	0.00
					Bradyrhizobium	1	0.00	0.00	0.00	0.11	0.04	0.07	0.05	0.00
	Proteobacteria			*Hyphomicrobiaceae*	*Rhodoplanes*	1	0.00	0.03	0.02	0.18	0.12	0.15	0.11	0.00
	Proteobacteria		*Rhodospirillales*	*Rhodospirillaceae*	*Azospirillum*	1	0.01	0.19	0.81	0.66	0.29	0.25	0.40	0.01
	Proteobacteria	*Betaproteobacteria*	*Burkholderiales*	*Burkholderiaceae*	*Burkholderia*	3	0.21	0.31	0.58	0.59	0.44	1.69	0.76	0.02
	Proteobacteria	*Gammaproteobacteria*	*Xanthomonadales*	*Xanthomonadaceae*	*Dyella*	1	0.08	0.20	0.10	0.69	0.21	0.70	0.40	0.00
OTUs without genus classification	*Acidobacteria*	*Acidobacteriia*	*Acidobacteriales*	*Koribacteraceae*	-	1	0.00	0.03	0.01	0.19	0.14	0.20	0.13	0.00
	Actinobacteria	*Actinobacteria*	*Actinomycetales*	-	-	2	0.01	0.23	0.65	0.39	0.60	0.20	0.41	0.03
				Actinospicaceae	-	2	0.07	1.17	0.65	0.36	1.51	0.77	0.93	0.04
	Bacteroidetes	*Saprospirae*	*Saprospirales*	*Chitinophagaceae*	-	1	0.08	0.06	0.07	0.07	0.04	0.08	0.06	0.01
	Proteobacteria	*Alphaproteobacteria*	*Rhizobiales*	-	-	1	0.00	0.02	0.00	0.07	0.05	0.11	0.05	0.00
			Rhizobiales	*Beijerinckiaceae*	-	1	0.11	0.09	0.15	0.09	0.06	0.11	0.09	0.01
				Methylocystaceae	-	2	0.14	0.65	0.78	0.60	0.58	0.68	0.64	0.11
			Rhodospirillales	*Acetobacteraceae*	-	2	0.00	0.04	0.00	0.13	0.18	0.20	0.13	0.00
				Rhodospirillaceae	-	5	0.34	0.26	0.54	0.57	0.50	0.50	0.48	0.04
		Betaproteobacteria	*Burkholderiales*	*Burkholderiaceae*	-	1	2.71	0.16	0.10	0.48	0.85	0.97	0.65	0.03
		Gammaproteobacteria	*Xanthomonadales*	*Sinobacteraceae*	-	4	0.48	0.56	1.09	0.89	1.16	0.93	0.94	0.14
					Sum	30	4.25	4.04	5.58	6.52	7.03	7.89	6.48	0.45

- Based on sparse partial least squares discriminant analysis (sPLSDA) on centered log ratio-transformed OTU counts
- Relative abundances are shaded by column from white to red representing lowest to highest values in the respective plant phyla.

Supplementary Table 6. List of root-associated taxa according to analysis

Indicator species analysis
p__Actinobacteria; c__Actinobacteria; o__Actinomycetales; f__Actinospicaceae; g__Actinospica
p__Actinobacteria; c__Actinobacteria; o__Actinomycetales; f__Actinosynnemataceae; g__Kutzneria
p__Actinobacteria; c__Actinobacteria; o__Actinomycetales; f__Micrococcaceae; g__Arthrobacter
p__Actinobacteria; c__Actinobacteria; o__Actinomycetales; f__Micromonosporaceae; g__Actinocatenispora
p__Actinobacteria; c__Actinobacteria; o__Actinomycetales; f__Mycobacteriaceae; g__Mycobacterium
p__Actinobacteria; c__Actinobacteria; o__Actinomycetales; f__Nocardiaceae; g__Nocardia
p__Actinobacteria; c__Actinobacteria; o__Actinomycetales; f__Pseudonocardiaceae; g__Amycolatopsis
p__Actinobacteria; c__Actinobacteria; o__Actinomycetales; f__Streptomycetaceae; g__Streptomyces
p__Actinobacteria; c__Actinobacteria; o__Actinomycetales; f__Streptosporangiaceae; g__Streptosporangium
p__Actinobacteria; c__Actinobacteria; o__Actinomycetales; f__Thermomonosporaceae; g__Actinomadura
p__Actinobacteria; c__Actinobacteria; o__Actinomycetales; f__Thermomonosporaceae; g__Spirillospora
p__Armatimonadetes; c__Fimbriimonadia; o__Fimbriimonadales; f__Fimbriimonadaceae; g__Fimbriimonas
p__Firmicutes; c__Bacilli; o__Bacillales; f__Bacillaceae; g__Bacillus
p__Proteobacteria; c__Alphaproteobacteria; o__Caulobacterales; f__Caulobacteraceae; g__Asticcacaulis
p__Proteobacteria; c__Alphaproteobacteria; o__Rhizobiales; f__Beijerinckiaceae; g__Beijerinckia
p__Proteobacteria; c__Alphaproteobacteria; o__Rhizobiales; f__Beijerinckiaceae; g__Methylocapsa
p__Proteobacteria; c__Alphaproteobacteria; o__Rhizobiales; f__Bradyrhizobiaceae; g__Afipia
p__Proteobacteria; c__Alphaproteobacteria; o__Rhizobiales; f__Bradyrhizobiaceae; g__Balneimonas
p__Proteobacteria; c__Alphaproteobacteria; o__Rhizobiales; f__Bradyrhizobiaceae; g__Bradyrhizobium
p__Proteobacteria; c__Alphaproteobacteria; o__Rhizobiales; f__Hyphomicrobiaceae; g__Rhodoplanes
p__Proteobacteria; c__Alphaproteobacteria; o__Rhizobiales; f__Phyllobacteriaceae; g__Mesorhizobium
p__Proteobacteria; c__Alphaproteobacteria; o__Rhizobiales; f__Phyllobacteriaceae; g__Nitratireductor
p__Proteobacteria; c__Alphaproteobacteria; o__Rhizobiales; f__Rhizobiaceae; g__Agrobacterium
p__Proteobacteria; c__Alphaproteobacteria; o__Rhizobiales; f__Rhizobiaceae; g__Rhizobium
p__Proteobacteria; c__Alphaproteobacteria; o__Rhizobiales; f__Rhizobiaceae; g__Shinella
p__Proteobacteria; c__Alphaproteobacteria; o__Rhizobiales; f__Xanthobacteraceae; g__Azorhizobium
p__Proteobacteria; c__Alphaproteobacteria; o__Rhizobiales; f__Xanthobacteraceae; g__Labrys
p__Proteobacteria; c__Alphaproteobacteria; o__Rhodospirillales; f__Acetobacteraceae; g__Acidiphilium
p__Proteobacteria; c__Alphaproteobacteria; o__Rhodospirillales; f__Acetobacteraceae; g__Acidisoma
p__Proteobacteria; c__Alphaproteobacteria; o__Rhodospirillales; f__Acetobacteraceae; g__Acidocella
p__Proteobacteria; c__Alphaproteobacteria; o__Rhodospirillales; f__Acetobacteraceae; g__Acidomonas
p__Proteobacteria; c__Alphaproteobacteria; o__Rhodospirillales; f__Acetobacteraceae; g__Rhodovastum

- Three analyses were used- indicator species analysis, Welch's t-test in STAMP and sparse partial least squares discriminant analysis (sPLSDA) in mixOmics
- Number of root-associated taxa- 86 in indicator species analysis, 75 in STAMP, 17 in sPLSDA
- Green shading indicates overlap in taxa between at least two analyses
- See Supplementary Fig. 9 for Venn diagram showing degree of overlap
- p: phylum; c: class; o: order; f: family; g: genus; s: species

p__Proteobacteria; c__Alphaproteobacteria; o__Rhodospirillales; f__Rhodospirillaceae; g__Azospirillum
p__Proteobacteria; c__Alphaproteobacteria; o__Rhodospirillales; f__Rhodospirillaceae; g__Telmatospirillum
p__Proteobacteria; c__Alphaproteobacteria; o__Sphingomonadales; f__Sphingomonadaceae; g__Kaistobacter
p__Proteobacteria; c__Alphaproteobacteria; o__Sphingomonadales; f__Sphingomonadaceae; g__Sphingobium
p__Proteobacteria; c__Alphaproteobacteria; o__Sphingomonadales; f__Sphingomonadaceae; g__Sphingomonas
p__Proteobacteria; c__Betaproteobacteria; o__Burkholderiales; f__Burkholderiaceae; g__Burkholderia
p__Proteobacteria; c__Betaproteobacteria; o__Burkholderiales; f__Burkholderiaceae; g__Pandoraea
p__Proteobacteria; c__Betaproteobacteria; o__Burkholderiales; f__Burkholderiaceae; g__Salinispora
p__Proteobacteria; c__Betaproteobacteria; o__Burkholderiales; f__Comamonadaceae; g__Rubrivivax
p__Proteobacteria; c__Betaproteobacteria; o__Burkholderiales; f__Oxalobacteraceae; g__Cupriavidus
p__Proteobacteria; c__Gammaproteobacteria; o__Xanthomonadales; f__Xanthomonadaceae; g__Dyella
p__Proteobacteria; c__Gammaproteobacteria; o__Xanthomonadales; f__Xanthomonadaceae; g__Luteibacter
p__Proteobacteria; c__Gammaproteobacteria; o__Xanthomonadales; f__Xanthomonadaceae; g__Rhodanobacter
p__Tenericutes; c__Mollicutes; o__Anaeroplasmatales; f__Anaeroplasmataceae; g__Asteroleplasma
p__Acidobacteria; c__Acidobacteriia; o__Acidobacteriales; f__Acidobacteriaceae; g__
p__Acidobacteria; c__Acidobacteriia; o__Acidobacteriales; f__Koribacteraceae; g__
p__Actinobacteria; c__Actinobacteria; o__Actinomycetales; f__; g__
p__Actinobacteria; c__Actinobacteria; o__Actinomycetales; f__Actinospicaceae; g__
p__Actinobacteria; c__Actinobacteria; o__Actinomycetales; f__Micromonosporaceae; g__
p__Actinobacteria; c__Actinobacteria; o__Actinomycetales; f__Pseudonocardiaceae; g__
p__Actinobacteria; c__Actinobacteria; o__Actinomycetales; f__Thermomonosporaceae; g__
p__Actinobacteria; c__Thermoleophilia; o__Gaiellales; f__Gaiellaceae; g__
p__Actinobacteria; c__Thermoleophilia; o__Solirubrobacterales; f__; g__
p__Actinobacteria; c__Thermoleophilia; o__Solirubrobacterales; f__Conexibacteraceae; g__
p__Armatimonadetes; c__Armatimonadia; o__FW68; f__; g__
p__Armatimonadetes; c__Chthonomonadetes; o__Chthonomonadales; f__Chthonomonadaceae; g__
p__Bacteroidetes; c__Saprospirae; o__Saprospirales; f__Chitinophagaceae; g__
p__Chloroflexi; c__Ktedonobacteria; o__JG30-KF-AS9; f__; g__
p__Chloroflexi; c__Ktedonobacteria; o__Ktedonobacterales; f__Ktedonobacteraceae; g__
p__Chloroflexi; c__Ktedonobacteria; o__Thermogemmatisporales; f__Thermogemmatisporaceae; g__
p__Gemmatimonadetes; c__Gemmatimonadetes; o__; f__; g__
p__Planctomycetes; c__Phycisphaerae; o__WD2101; f__; g__
p__Proteobacteria; c__Alphaproteobacteria; o__; f__; g__
p__Proteobacteria; c__Alphaproteobacteria; o__Caulobacterales; f__Caulobacteraceae; g__

p__Proteobacteria; c__Alphaproteobacteria; o__Ellin329; f__; g__
p__Proteobacteria; c__Alphaproteobacteria; o__Rhizobiales; f__; g__
p__Proteobacteria; c__Alphaproteobacteria; o__Rhizobiales; f__Beijerinckiaceae; g__
p__Proteobacteria; c__Alphaproteobacteria; o__Rhizobiales; f__Bradyrhizobiaceae; g__
p__Proteobacteria; c__Alphaproteobacteria; o__Rhizobiales; f__Hyphomicrobiaceae; g__
p__Proteobacteria; c__Alphaproteobacteria; o__Rhizobiales; f__Methylocystaceae; g__
p__Proteobacteria; c__Alphaproteobacteria; o__Rhizobiales; f__Phyllobacteriaceae; g__
p__Proteobacteria; c__Alphaproteobacteria; o__Rhizobiales; f__Rhizobiaceae; g__
p__Proteobacteria; c__Alphaproteobacteria; o__Rhodospirillales; f__Acetobacteraceae; g__
p__Proteobacteria; c__Alphaproteobacteria; o__Rhodospirillales; f__Rhodospirillaceae; g__
p__Proteobacteria; c__Alphaproteobacteria; o__Sphingomonadales; f__Sphingomonadaceae; g__
p__Proteobacteria; c__Betaproteobacteria; o__Burkholderiales; f__Burkholderiaceae; g__
p__Proteobacteria; c__Betaproteobacteria; o__Burkholderiales; f__Comamonadaceae; g__
p__Proteobacteria; c__Betaproteobacteria; o__Burkholderiales; f__Oxalobacteraceae; g__
p__Proteobacteria; c__Gammaproteobacteria; o__Acidithiobacillales; f__; g__
p__Proteobacteria; c__Gammaproteobacteria; o__Xanthomonadales; f__Sinobacteraceae; g__
p__Proteobacteria; c__Gammaproteobacteria; o__Xanthomonadales; f__Xanthomonadaceae; g__
p__TM7; c__TM7-1; o__; f__; g__
p__Verrucomicrobia; c__Methylacidiphilae; o__Methylacidiphilales; f__; g__
p__WPS-2; c__; o__; f__; g__

Welch's t-test in STAMP
p__Actinobacteria; c__Actinobacteria; o__Actinomycetales; f__Actinospicaceae; g__Actinospica
p__Actinobacteria; c__Actinobacteria; o__Actinomycetales; f__Actinosynnemataceae; g__Kutzneria
p__Actinobacteria; c__Actinobacteria; o__Actinomycetales; f__Micrococcaceae; g__Arthrobacter
p__Actinobacteria; c__Actinobacteria; o__Actinomycetales; f__Micromonosporaceae; g__Actinocatenispora
p__Actinobacteria; c__Actinobacteria; o__Actinomycetales; f__Mycobacteriaceae; g__Mycobacterium
p__Actinobacteria; c__Actinobacteria; o__Actinomycetales; f__Streptomycetaceae; g__Streptomyces
p__Actinobacteria; c__Actinobacteria; o__Actinomycetales; f__Streptosporangiaceae; g__Streptosporangium
p__Actinobacteria; c__Actinobacteria; o__Actinomycetales; f__Thermomonosporaceae; g__Actinomadura
p__Actinobacteria; c__Actinobacteria; o__Actinomycetales; f__Thermomonosporaceae; g__Spirillospora
p__Armatimonadetes; c__Fimbriimonadia; o__Fimbriimonadales; f__Fimbriimonadaceae; g__Fimbriimonas
p__Firmicutes; c__Bacilli; o__Bacillales; f__Bacillaceae; g__Bacillus
p__Proteobacteria; c__Alphaproteobacteria; o__Caulobacterales; f__Caulobacteraceae; g__Asticcacaulis
p__Proteobacteria; c__Alphaproteobacteria; o__Rhizobiales; f__Beijerinckiaceae; g__Beijerinckia
p__Proteobacteria; c__Alphaproteobacteria; o__Rhizobiales; f__Beijerinckiaceae; g__Methylocapsa
p__Proteobacteria; c__Alphaproteobacteria; o__Rhizobiales; f__Beijerinckiaceae; g__Methylovirgula
p__Proteobacteria; c__Alphaproteobacteria; o__Rhizobiales; f__Bradyrhizobiaceae; g__Afipia
p__Proteobacteria; c__Alphaproteobacteria; o__Rhizobiales; f__Bradyrhizobiaceae; g__Balneimonas
p__Proteobacteria; c__Alphaproteobacteria; o__Rhizobiales; f__Bradyrhizobiaceae; g__Bradyrhizobium
p__Proteobacteria; c__Alphaproteobacteria; o__Rhizobiales; f__Hyphomicrobiaceae; g__Rhodoplanes
p__Proteobacteria; c__Alphaproteobacteria; o__Rhizobiales; f__Phyllobacteriaceae; g__Mesorhizobium
p__Proteobacteria; c__Alphaproteobacteria; o__Rhizobiales; f__Phyllobacteriaceae; g__Nitratireductor
p__Proteobacteria; c__Alphaproteobacteria; o__Rhizobiales; f__Rhizobiaceae; g__Agrobacterium
p__Proteobacteria; c__Alphaproteobacteria; o__Rhizobiales; f__Rhizobiaceae; g__Rhizobium
p__Proteobacteria; c__Alphaproteobacteria; o__Rhizobiales; f__Rhizobiaceae; g__Shinella
p__Proteobacteria; c__Alphaproteobacteria; o__Rhizobiales; f__Xanthobacteraceae; g__Labrys
p__Proteobacteria; c__Alphaproteobacteria; o__Rhodospirillales; f__Acetobacteraceae; g__Acidiphilium
p__Proteobacteria; c__Alphaproteobacteria; o__Rhodospirillales; f__Acetobacteraceae; g__Acidisoma
p__Proteobacteria; c__Alphaproteobacteria; o__Rhodospirillales; f__Acetobacteraceae; g__Acidocella
p__Proteobacteria; c__Alphaproteobacteria; o__Rhodospirillales; f__Acetobacteraceae; g__Acidomonas
p__Proteobacteria; c__Alphaproteobacteria; o__Rhodospirillales; f__Rhodospirillaceae; g__Azospirillum
p__Proteobacteria; c__Alphaproteobacteria; o__Rhodospirillales; f__Rhodospirillaceae; g__Telmatospirillum
p__Proteobacteria; c__Alphaproteobacteria; o__Sphingomonadales; f__Sphingomonadaceae; g__Kaistobacter

p__Proteobacteria; c__Alphaproteobacteria; o__Sphingomonadales; f__Sphingomonadaceae; g__Sphingomonas
p__Proteobacteria; c__Betaproteobacteria; o__Burkholderiales; f__Burkholderiaceae; g__Burkholderia
p__Proteobacteria; c__Betaproteobacteria; o__Burkholderiales; f__Burkholderiaceae; g__Salinispora
p__Proteobacteria; c__Betaproteobacteria; o__Burkholderiales; f__Comamonadaceae; g__Rubrivivax
p__Proteobacteria; c__Betaproteobacteria; o__Burkholderiales; f__Oxalobacteraceae; g__Cupriavidus
p__Proteobacteria; c__Gammaproteobacteria; o__Xanthomonadales; f__Xanthomonadaceae; g__Dyella
p__Proteobacteria; c__Gammaproteobacteria; o__Xanthomonadales; f__Xanthomonadaceae; g__Rhodanobacter
p__Tenericutes; c__Mollicutes; o__Anaeroplasmatales; f__Anaeroplasmataceae; g__Asteroleplasma
p__Acidobacteria; c__Acidobacteriia; o__Acidobacteriales; f__Acidobacteriaceae; g__
p__Acidobacteria; c__Acidobacteriia; o__Acidobacteriales; f__Koribacteraceae; g__
p__Actinobacteria; c__Actinobacteria; o__Actinomycetales; f__; g__
p__Actinobacteria; c__Actinobacteria; o__Actinomycetales; f__Actinospicaceae; g__
p__Actinobacteria; c__Actinobacteria; o__Actinomycetales; f__Micromonosporaceae; g__
p__Actinobacteria; c__Actinobacteria; o__Actinomycetales; f__Pseudonocardiaceae; g__
p__Actinobacteria; c__Actinobacteria; o__Actinomycetales; f__Thermomonosporaceae; g__
p__Actinobacteria; c__Thermoleophilia; o__Gaiellales; f__Gaiellaceae; g__
p__Actinobacteria; c__Thermoleophilia; o__Solirubrobacterales; f__; g__
p__Actinobacteria; c__Thermoleophilia; o__Solirubrobacterales; f__Conexibacteraceae; g__
p__Armatimonadetes; c__Armatimonadia; o__FW68; f__; g__
p__Bacteroidetes; c__Saprospirae; o__Saprospirales; f__Chitinophagaceae; g__
p__Chloroflexi; c__Ktedonobacteria; o__JG30-KF-AS9; f__; g__
p__Chloroflexi; c__Ktedonobacteria; o__Ktedonobacterales; f__Ktedonobacteraceae; g__
p__Gemmatimonadetes; c__Gemmatimonadetes; o__; f__; g__
p__Planctomycetes; c__Phycisphaerae; o__WD2101; f__; g__
p__Proteobacteria; c__Alphaproteobacteria; o__Caulobacterales; f__Caulobacteraceae; g__
p__Proteobacteria; c__Alphaproteobacteria; o__Ellin329; f__; g__
p__Proteobacteria; c__Alphaproteobacteria; o__Rhizobiales; f__; g__
p__Proteobacteria; c__Alphaproteobacteria; o__Rhizobiales; f__Beijerinckiaceae; g__
p__Proteobacteria; c__Alphaproteobacteria; o__Rhizobiales; f__Bradyrhizobiaceae; g__
p__Proteobacteria; c__Alphaproteobacteria; o__Rhizobiales; f__Methylocystaceae; g__
p__Proteobacteria; c__Alphaproteobacteria; o__Rhizobiales; f__Phyllobacteriaceae; g__
p__Proteobacteria; c__Alphaproteobacteria; o__Rhizobiales; f__Rhizobiaceae; g__
p__Proteobacteria; c__Alphaproteobacteria; o__Rhodospirillales; f__Acetobacteraceae; g__
p__Proteobacteria; c__Alphaproteobacteria; o__Rhodospirillales; f__Rhodospirillaceae; g__

p__Proteobacteria; c__Alphaproteobacteria; o__Sphingomonadales; f__Sphingomonadaceae; g__
p__Proteobacteria; c__Betaproteobacteria; o__Burkholderiales; f__Burkholderiaceae; g__
p__Proteobacteria; c__Betaproteobacteria; o__Burkholderiales; f__Comamonadaceae; g__
p__Proteobacteria; c__Betaproteobacteria; o__Burkholderiales; f__Oxalobacteraceae; g__
p__Proteobacteria; c__Gammaproteobacteria; o__Xanthomonadales; f__Sinobacteraceae; g__
p__Proteobacteria; c__Gammaproteobacteria; o__Xanthomonadales; f__Xanthomonadaceae; g__
p__TM7; c__TM7-1; o__; f__; g__
p__Verrucomicrobia; c__Methylacidiphilae; o__Methylacidiphilales; f__; g__
p__WPS-2; c__; o__; f__; g__

sPLSDA in mixOmics

p__Proteobacteria; c__Alphaproteobacteria; o__Rhizobiales; f__Bradyrhizobiaceae; g__Afipia
p__Proteobacteria; c__Alphaproteobacteria; o__Rhizobiales; f__Bradyrhizobiaceae; g__Bradyrhizobium
p__Proteobacteria; c__Alphaproteobacteria; o__Rhizobiales; f__Hyphomicrobiaceae; g__Rhodoplanes
p__Proteobacteria; c__Alphaproteobacteria; o__Rhodospirillales; f__Rhodospirillaceae; g__Azospirillum
p__Proteobacteria; c__Betaproteobacteria; o__Burkholderiales; f__Burkholderiaceae; g__Burkholderia
p__Proteobacteria; c__Gammaproteobacteria; o__Xanthomonadales; f__Xanthomonadaceae; g__Dyella
p__Acidobacteria; c__Acidobacteriia; o__Acidobacteriales; f__Koribacteraceae; g__
p__Actinobacteria; c__Actinobacteria; o__Actinomycetales; f__; g__
p__Actinobacteria; c__Actinobacteria; o__Actinomycetales; f__Actinospicaceae; g__
p__Bacteroidetes; c__Saprospirae; o__Saprospirales; f__Chitinophagaceae; g__
p__Proteobacteria; c__Alphaproteobacteria; o__Rhizobiales; f__; g__
p__Proteobacteria; c__Alphaproteobacteria; o__Rhizobiales; f__Beijerinckiaceae; g__
p__Proteobacteria; c__Alphaproteobacteria; o__Rhizobiales; f__Methylocystaceae; g__
p__Proteobacteria; c__Alphaproteobacteria; o__Rhodospirillales; f__Acetobacteraceae; g__
p__Proteobacteria; c__Alphaproteobacteria; o__Rhodospirillales; f__Rhodospirillaceae; g__
p__Proteobacteria; c__Betaproteobacteria; o__Burkholderiales; f__Burkholderiaceae; g__
p__Proteobacteria; c__Gammaproteobacteria; o__Xanthomonadales; f__Sinobacteraceae; g__

Supplementary Table 7. Cooloola core root microbiome taxa summarized by lowest assigned taxonomic rank (based on indicator species analysis on rarefaction normalized OTU counts, 1000 reads per sample)

	Taxonomy						Average relative abundance (%)								Also core in:				
	Phylum	Class	Order	Family	Genus	Number of OTUs represented	Lycopod	Ferns	Gymno-sperms	Magnoliids	Eudicots	Monocots	All roots	All soils	sugar-cane	*A. thaliana*	barley	grape-vine	maize
OTUs with genus classification	*Actinobacteria*	*Actinobacteria*	*Actinomycetales*	*Actinospicaceae*	*Actinospica*	4	0.03	0.27	0.20	0.57	0.51	0.24	0.38	0.03					
				Microbacteriaceae	*Salinibacterium*	2	0.00	0.01	0.13	0.03	0.00	0.04	0.03	0.00			Y		
				Mycobacteriaceae	*Mycobacterium*	40	0.15	1.00	1.05	1.02	1.52	1.66	1.31	0.52	Y				
				Streptomycetaceae	*Streptomyces*	1	0.14	0.02	0.01	0.01	0.00	0.01	0.01	0.00	Y	Y		Y	Y
	Armatimonadetes	*Fimbriimonadia*	*Fimbriimonadales*	*Fimbriimonadaceae*	*Fimbriimonas*	4	0.03	0.03	0.08	0.13	0.05	0.06	0.07	0.01	Y			Y	
	Proteobacteria	*Alphaproteobacteria*	*Caulobacterales*	*Caulobacteraceae*	*Asticcacaulis*	4	0.18	0.16	0.12	0.33	0.09	0.15	0.16	0.02	Y	Y		Y	
			Rhizobiales	*Beijerinckiaceae*	*Methylocapsa*	2	0.00	0.04	0.43	0.08	0.11	0.10	0.13	0.03					
				Bradyrhizobiaceae	*Afipia*	4	0.00	0.04	0.03	0.54	0.37	0.37	0.31	0.00	Y				
					Bradyrhizobium	21	2.52	5.60	7.42	6.93	6.00	5.86	6.18	3.16	Y	Y		Y	
				Hyphomicrobiaceae	*Hyphomicrobium*	2	0.14	0.04	0.04	0.19	0.05	0.08	0.08	0.04	Y	Y		Y	
					Rhodoplanes	33	0.27	1.31	0.84	1.73	1.44	1.07	1.30	0.53	Y	Y		Y	
				Phyllobacteriaceae	*Mesorhizobium*	1	0.00	0.09	0.18	0.23	0.11	0.06	0.12	0.02	Y	Y	Y	Y	
					Nitratireductor	2	0.00	0.00	0.00	0.14	0.03	0.03	0.04	0.00					
				Rhizobiaceae	*Agrobacterium*	1	0.00	0.01	0.06	0.03	0.09	0.10	0.07	0.00	Y	Y	Y	Y	Y
					Rhizobium	5	0.54	0.04	0.43	0.66	0.07	0.26	0.27	0.00	Y	Y	Y	Y	Y
					Shinella	1	0.04	0.03	0.37	0.03	0.08	0.05	0.08	0.00					
				Xanthobacteraceae	*Labrys*	6	0.30	0.06	0.17	0.21	0.03	0.09	0.10	0.02	Y			Y	
			Rhodospirillales	*Acetobacteraceae*	*Acidisoma*	6	0.10	0.09	0.20	0.26	0.24	0.09	0.18	0.03	Y				
					Acidocella	8	0.58	0.48	0.31	0.33	0.81	0.16	0.45	0.13					
				Rhodospirillaceae	*Azospirillum*	11	0.07	0.08	0.54	0.31	0.13	0.13	0.20	0.01	Y				
					Telmatospirillum	8	1.24	0.26	0.16	1.11	0.25	0.38	0.45	0.07	Y				
			Sphingomonadales	*Sphingomonadaceae*	*Sphingomonas*	5	0.44	0.03	0.18	0.18	0.18	0.07	0.14	0.02	Y	Y	Y	Y	
		Betaproteobacteria	*Burkholderiales*	*Burkholderiaceae*	*Burkholderia*	48	2.87	0.43	0.60	0.88	0.83	1.59	0.97	0.09	Y	Y			Y
					Salinispora	12	2.65	0.42	0.80	0.47	0.43	0.66	0.57	0.12	Y				
				Comamonadaceae	*Rubrivivax*	6	0.22	0.03	0.09	0.07	0.14	0.12	0.10	0.00	Y	Y	Y	Y	
		Gammaproteobacteria	*Xanthomonadales*	*Xanthomonadaceae*	*Dyella*	10	0.15	0.22	0.14	0.80	0.33	1.45	0.66	0.01	Y				
	Tenericutes	*Mollicutes*	*Anaeroplasmatales*	*Anaeroplasmataceae*	*Asteroleplasma*	19	0.00	0.04	0.55	0.20	0.19	0.37	0.25	0.00					

- Relative abundances are shaded by column from white to red representing lowest to highest values in the respective plant phyla.

Supplementary Table 7 continued – OTUs without genus classification

	Taxonomy						Average relative abundance (%)								Also core in:				
	Phylum	Class	Order	Family	Genus	Number of OTUs represented	Lycopod	Ferns	Gymno-sperms	Magnoliids	Eudicots	Monocots	All roots	All soils	sugar-cane	*A. thaliana*	barley	grape-vine	maize
Otus without genus classification	*Acidobacteria*	*Acidobacteriia*	*Acidobacteriales*	*Acidobacteriaceae*	-	40	4.20	3.46	3.54	2.65	3.51	2.79	3.18	1.27	Y				
				Koribacteraceae	-	20	0.27	0.12	0.06	0.39	0.29	0.33	0.27	0.01				Y	
	Actinobacteria	*Acidimicrobiia*	*Acidimicrobiales*	-	-	7	0.00	0.02	0.00	0.03	0.06	0.12	0.06	0.00				Y	
		Actinobacteria	*Actinomycetales*	-	-	70	0.27	1.45	2.31	1.48	2.17	0.98	1.63	0.36	Y				
				Actinospicaceae	-	22	0.04	0.61	0.64	0.45	0.77	0.65	0.63	0.03	Y				
				Micromonosporaceae	-	3	0.08	0.20	0.30	0.22	0.01	0.13	0.14	0.02	Y			Y	
				Pseudonocardiaceae	-	3	0.00	0.01	0.25	0.35	0.16	0.17	0.18	0.05	Y			Y	
		Thermoleophilia	*Gaiellales*	*Gaiellaceae*	-	5	0.00	0.00	0.01	0.05	0.02	0.11	0.04	0.00	Y			Y	
			Solirubrobacterales	-	-	4	0.00	0.00	0.02	0.16	0.09	0.11	0.09	0.00	Y			Y	
				Conexibacteraceae	-	9	0.08	0.30	0.20	0.41	0.80	0.41	0.48	0.13					
				Solirubrobacteraceae	-	3	0.14	0.12	0.03	0.06	0.04	0.04	0.05	0.00					
	Armatimonadetes	*Armatimonadia*	*FW68*	-	-	9	0.40	0.13	0.09	0.08	0.09	0.11	0.10	0.01	Y				
	Bacteroidetes	*Saprospirae*	*Saprospirales*	*Chitinophagaceae*	-	11	0.53	0.32	0.35	0.44	0.22	0.38	0.33	0.07	Y			Y	
	Chloroflexi	*Ktedonobacteria*	*Ktedonobacterales*	*Ktedonobacteraceae*	-	15	0.00	0.17	0.30	0.03	0.47	0.17	0.24	0.01	Y			Y	
			Thermogemmatisporales	*Thermogemmatisporaceae*	-	2	0.00	0.04	0.15	0.00	0.04	0.00	0.03	0.00	Y				
	Gemmatimonadetes	*Gemmatimonadetes*	-	-	-	3	0.00	0.11	0.15	0.11	0.12	0.11	0.12	0.02				Y	
	Planctomycetes	*Phycisphaerae*	*WD2101*	-	-	3	0.03	0.11	0.11	0.07	0.08	0.05	0.08	0.03				Y	
	Proteobacteria	*Alphaproteobacteria*	*Caulobacterales*	*Caulobacteraceae*	-	24	1.22	0.23	0.36	0.52	0.55	0.62	0.51	0.10	Y	Y		Y	
			Ellin329	-	-	9	0.08	0.10	0.13	0.16	0.13	0.08	0.12	0.02	Y			Y	
			Rhizobiales	-	-	14	0.17	0.09	0.13	0.17	0.14	0.21	0.15	0.01	Y	Y	Y	Y	
				Beijerinckiaceae	-	9	0.21	0.18	0.30	0.20	0.15	0.24	0.20	0.02					
				Bradyrhizobiaceae	-	25	0.25	0.61	1.77	0.78	0.67	0.99	0.87	0.24	Y			Y	
				Methylocystaceae	-	32	1.39	1.53	1.33	1.42	1.49	1.60	1.49	0.67	Y				
				Rhizobiaceae	-	4	0.24	0.12	0.38	0.15	0.05	0.19	0.15	0.02	Y	Y	Y	Y	
			Rhodospirillales	*Acetobacteraceae*	-	48	0.89	0.40	0.85	0.83	0.69	0.47	0.64	0.17	Y				
				Rhodospirillaceae	-	83	3.31	2.32	2.47	2.83	2.02	2.00	2.28	0.67	Y			Y	
		Betaproteobacteria	*Burkholderiales*	*Burkholderiaceae*	-	4	1.50	0.09	0.10	0.35	0.79	0.73	0.52	0.02					
				Comamonadaceae	-	8	0.18	0.05	0.29	0.16	0.13	0.31	0.19	0.02	Y	Y	Y	Y	Y
		Gammaproteobacteria	*Xanthomonadales*	*Sinobacteraceae*	-	34	1.62	1.02	1.90	1.86	2.00	1.53	1.69	0.44	Y	Y		Y	
				Xanthomonadaceae	-	14	0.46	0.31	0.32	0.45	0.33	0.50	0.39	0.04	Y	Y	Y	Y	
	Verrucomicrobia	*Methylacidiphilae*	*Methylacidiphilales*	-	-	5	0.19	0.08	0.20	0.15	0.06	0.08	0.10	0.03	Y			Y	
	WPS-2	-	-	-	-	6	0.09	0.11	0.24	0.70	0.26	0.39	0.35	0.11	Y				
					Sum:	814	30.5	25.2	34.3	35.2	32.5	31.9	31.9	9.5	Australia	Germany		America	
					Sum including low abundance OTUs:	959	30.8	26.0	35.4	36.6	33.9	33.2	33.2	9.6					
